# Scrotal abscess as a complication of perforated appendicitis: A case report and review of the literature

**DOI:** 10.1186/1757-1626-1-165

**Published:** 2008-09-19

**Authors:** Mohammad M Saleem

**Affiliations:** 1 The Department of Pediatric Surgery, Jordan University Hospital, University of Jordan, Amman, Jordan

## Abstract

**Introduction:**

Abscess formation following appendectomy is well known, especially when complicated by perforation. Infectious complications are the most common. Intraabdominal abscess formation, Pelvic abscess and wound infection are the most common sites of infection. Scrotal abscess following acute perforated appendicitis is very rare.

**Cases presentation:**

We report two cases of scrotal abscess following perforated appendicitis, one was 1983 and the other in 1997. The first patient developed acute left hemiscrotum two weeks following correction of a tetralogy of Fallot that was diagnosed since infancy. Scrotal drainage as well as open appendectomy and abdominal drainage were followed by uneventful recovery. Six weeks later left groin exploration revealed patent processus vaginalis which was ligated. The second patient developed redness, swelling and pain in his left hemiscrotum 10 days after open appendectomy for perforated appendicitis. Groin exploration, ligation of a PPV and scrotal drainage was made. Recent reports on the subject, review of the literature, the rarity of the complication, and the possible association with recent introduction of laparoscopic appendectomy.

**Conclusion:**

Acute scrotal swelling is frequently a surgical emergency. Developing in the post-operative period is no exception. Symptoms and signs may be hampered by analgesia, pain, and antibiotics, usually administered in this period. Reporting these rare complications following such a common procedure, especially now a day in the era of laparoscopic surgery. Only high degree of suspicion and vigilant intervention will accomplish a safe diagnosis and treatment. The appropriate time and approach to both abscess and PPV is still controversial. Until enough case reports treatment is to be individualized.

## Introduction

The scrotum develops as a part of the abdominal cavity, and the processus vaginalis remains patent 80–90% of newborns, and gradually declines to 15–37% during adulthood. In recent years several case reports of intraabdominal pathologies finding their way into the scrotum especially during peritoneal dialysis, or after placement of ventriculo-peritoneal shunt. The present cases and the literature review show that intraabdominal purulent collection may find its way to the scrotum via a PPV. The sine qua non of all these cases is the PPV.

## Cases presentation

### Case No 1

**Figure 1:**
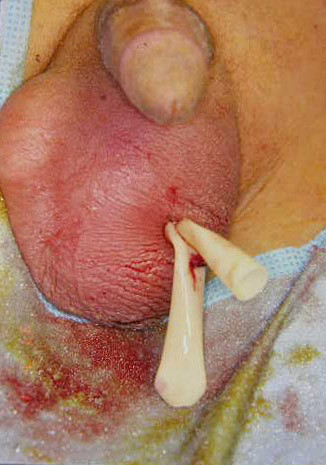
Scrotal drainage following groin exploration.

A 10-year-old male presented with 3 days history of abdominal pain, Anorehxia, and vomiting, admitted with the diagnosis of acute appendicitis. Abdominal examination revealed tenderness and rebound tenderness in the right lower quadrant of the abdomen. Temperature was 37.9°C and leucocytosis was 16,000 mm3, with left shift. At surgery acute perforated appendicitis was removed and because of the abscess cavity and difficulty in removing the appendix drainage was instituted, the peritoneal fluid was sent for culture and broad-spectrum antibiotic coverage was started. On post-operative day 5 the patient complained of swelling and redness of his left scrotal sac. There was minimal pain, US examination and Doppler US revealed a normal testis with normal blood flow, the fluid to contain debris suspected to be an abscess. Groin exploration revealed an abscess in a patent prcessus vaginalis (PPV). The PPV was ligated and the abscess was drained via the scrotum as shown in figure (1). The culture from the scrotal fluid did not grow any organism, while the peritoneal fluid grew gram negative E-coli. They were removed when the drainage stopped and the patient made a good recovery.

### Case No 2

**Figure 2:**
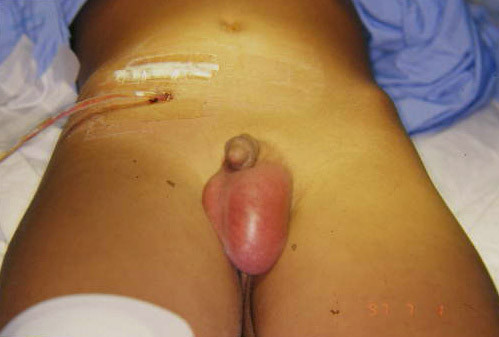
**Pelvic drainage after appendectomy. **Notice the scrotal swelling and redness of the left scrotal sac.

A 4-year-old male complained of lower abdominal pain, fever and swelling of the left scrotal sac of two days duration. He is known to have congenital heart disease, Tetralogy of Fallot, for which he underwent surgical correction two weeks earlier. Examination revealed a swollen left scrotal sac with tenderness in the scrotum, groin and lower abdomen. Temperature was 39.2°C, WBC 14,200 mm3. Diagnosis of acute scrotum was entertained and scrotal exploration was made. There was purulent scrotal fluid but the testis and epidydimis were normal. The fluid was thought to come from the abdomen as by pressing the lower abdomen the fluid was coming more. The scrotum was drained. On exploring the abdomen acute perforated pelvic appendicitis in a situs inversus situation was encountered. Appendectomy and drainage of the pelvis was performed Figure (2). Cultures from both the scrotum and peritoneum grew the same organism, Enterobacter. The drains were removed in one week. Antibiotic coverage was continued for two weeks and the patient made uneventful recovery. Six weeks later exploration of the groin revealed PPV, which was ligated.

## Discussion

The relationship between the scrotum and the peritoneal cavity has long been known. Examination of the scrotum is part of the abdominal examination. Patent processus vaginalis (PPV) is thought to remain patent in 15–37% of people beyond neonatal period. Suppuration following acute appendicitis is well known and occurs in 3–9% following acute appendicitis [1]. Abscess formation commonly occurs in the pelvis, between intestinal loops and in the subphrenic space. Scrotum as an extension of the peritoneal cavity is very rarely considered as a site of abscess formation following appendicitis. Only recently this subject has been recognized and reported. About 30 pediatric cases have been reported in the literature including neonates and adolescents. There were two previous reviews [2,3]. Ours is the largest one [2-17]. We have reviewed 22 cases in which enough information was available (Table 1). Three were neonates and three were adolescents [4-8]. All were males except one female 3 years old developed an abscess in the labial fold, seven days following appendectomy for perforated appendicitis and had a patent processus vaginalis. This is the only female reported with this complication [9]. Fifteen patients had a PPV and 14 required secondary operation following their primary appendectomy procedure. These were 1–10 days postoperatively. Five patients had their primary surgery inguino-scrotal to manage both the appendectomy as well as the drainage of the scrotal suppuration [4-6,10-12]. It is postulated that laparoscopic appendectomy with insufflations of the peritoneal cavity may potentiate the patent processus vaginalis and increase the incidence of scrotal contamination by peritoneal fluid. Three cases were following laparoscopic appendectomy [3,7,8]. Sixteen of the reported cases and our two cases were not following laparoscopic appendectomy. Five scrotal abscesses were approached through the scrotum and three were approached inguinally. Six cases including one of ours the inguino-scrotal exploration preceded the appendectomy procedure, and five of them required no further procedure [4-6,10-12]. Absence of history of inguinal hernia or hydrocele was common to all patients including ours; the timing of abscess development was between the 1^st ^and the 10th day following appendectomy. Presentation was scrotal swelling and redness in all the reported cases. Pain was not the outstanding feature and if this was present it was not excruciating, as this happen in the cases of torsion of the testis, or strangulated hernia. All the patients were explored with the provisional diagnosis of inguinal hernia strangulation or incarceration. There is a reported case of testicular damage the mechanism of which is obscured [13]. In our cases the PPV were wide open at the time of abscess formation. Ligation of the PPV was performed in the first presented case during the emergency groin exploration while in the second case this was accomplished six weeks later after appendectomy.

**Table 1:** a literature cases of scrotal abscess in children and adolescents as a complication of perforated appendicitis

**Author**	**Age **(years)	**Surgery **(Primary)	**Surgery **(Secondary)	**PPV**
Thakur(2001)^3 ^Case1	9	Open appy,	Inguinal I&D, (4)	+
Case2	7	Lap appy	Inguinal & scrotal I&D (10)	+
Dessanti (1995)^4^	18 Days	Rt. inguinal herniotomy, appy	No further surgery	+
Ibrahim & Malki (2000)^5^	22 Days	Scrotal exploration	No further surgery	-
Martin (2001)^6^	4 Days	Groin exploration, Open appy, herniotomy	No further surgery	+
Lantsberg & Mor (1997)^7^	20	Lap appy	Scrotal I&D (1)	-
Kollias & Gallery (1996)^8^	17	Lap appy	Scrotal I&D (4)	-
Bengol-kologlu (2006)^9^	3 Female	Open appy	Groin exploration (7)	+
	4	Open appy	Groin exploration (5)	+
	7	Open appy	Groin exploration (4)	-
	7	Open appy	Groin exploration (5)	-
	9	Open appy	Groin exploration (5)	-
Sharma (2004)^10^	6	Rt. Inguinal, herniotomy, Open appy	No further surgery	+
Singh (2003)^11^	2	Rt. Inguinal I&D, Open appy	No further surgery	+
Satchthananda (2000)^12^	3	Open appy	No further surgery	+
Robertson (1993)^13^	7	Open appy	Scrotal exploration and orchiectomy	+
Pal KMI(1995)^14^	8	Open appy	Scrotal I&D	-
Gan&Sweeny(1992)^15^	8	Open appy	Inguinal I&D (2)	-
McKerrow & Thomson(1982)^16^	9	Open appy	Scrotal I&D (2)	-
Lee Yung-chin(2003)^17^	19	Open appy	Scrotal exploration (3) Drainage of retroperitoneal abscess	-
Present cases Case 1	10	Open appy,	Inguinal I&D (5)	+
Case 2	4	Scrotal exploration	Herniotomy later	+

Total	22			

Key: Appy: appendectomy, Lap: laparoscopic, PPV: patent processus vaginalis, postoperative days in parenthesis.

## Conclusion

Acute appendicitis with scrotal involvement in children may present with scrotal abscess or with appearance of acute scrotum mimicking acute testicular torsion or incarcerated inguinal hernia. This may precede the diagnosis of acute appendicitis or as a postoperative complication of suppurative or perforated appendicitis.

Acute scrotal swelling is frequently a surgical emergency. Developing in the post-operative period is no exception. Analgesia, and antibiotics may hamper symptoms and signs, as these are usually administered the postoperative period. The unusual complication might follow common surgical procedures such as appendectomy, especially in laparoscopic surgery.

Only high degree of suspicion and vigilant intervention will accomplish a safe diagnosis and treatment. The appropriate time and approach to both abscess and PPV is still controversial. Until enough case reports treatment is to be individualized.

## Abbreviations

PPV: patent processus vaginalis; US: Ultrasound.

## Consent

Written informed consent was obtained from the patients' parents for publication of this case report and accompanying images. A copy of the written consent is available for review by the Editor-in-Chief of this journal.

## Competing interests

The author declares that they have no competing interests.

## Authors' contributions

This author has managed this case and prepared the manuscript.
